# Polygenic risk scores: from research tools to clinical instruments

**DOI:** 10.1186/s13073-020-00742-5

**Published:** 2020-05-18

**Authors:** Cathryn M. Lewis, Evangelos Vassos

**Affiliations:** 1 Social, Genetic and Developmental Psychiatry Centre, Institute of Psychiatry, Psychology & Neuroscience, King’s College London, de Crespigny Park, London, SE5 8AF UK; 2 Department of Medical and Molecular Genetics, Faculty of Life Sciences and Medicine, King’s College London, London, UK

**Keywords:** Genetics, Common disorders, Polygenic risk scores, Prediction, Pharmacogenetics, Risk

## Abstract

Genome-wide association studies have shown unequivocally that common complex disorders have a polygenic genetic architecture and have enabled researchers to identify genetic variants associated with diseases. These variants can be combined into a polygenic risk score that captures part of an individual’s susceptibility to diseases. Polygenic risk scores have been widely applied in research studies, confirming the association between the scores and disease status, but their clinical utility has yet to be established. Polygenic risk scores may be used to estimate an individual’s lifetime genetic risk of disease, but the current discriminative ability is low in the general population. Clinical implementation of polygenic risk score (PRS) may be useful in cohorts where there is a higher prior probability of disease, for example, in early stages of diseases to assist in diagnosis or to inform treatment choices. Important considerations are the weaker evidence base in application to non-European ancestry and the challenges in translating an individual’s PRS from a percentile of a normal distribution to a lifetime disease risk. In this review, we consider how PRS may be informative at different points in the disease trajectory giving examples of progress in the field and discussing obstacles that need to be addressed before clinical implementation.

## Background

Over the last decade, genome-wide association studies (GWAS) have uncovered the contribution of inherited variants to common complex disorders. Our current understanding is that most non-communicable disorders with a major public health impact have a genetic underpinning that is highly polygenic, comprising hundreds or thousands of genetic variants (or polymorphisms), each having a small effect on disease risk. Each genetic variant associated with a disease is valuable in indicating a gene or pathway of biological relevance to the disorder, but there are also expectations that the genetic data could be used to predict disease risk, with potential clinical utility.

In a polygenic disorder, a single variant is not informative for assessing disease risk. Instead, a genetic loading conferred by the combined set of risk variants is necessary to obtain a measure that has sufficient information to identify those at high risk. There are many possible approaches to combine information across loci; the genetic risk is most often assessed through the polygenic risk score (PRS), a weighted sum of the number of risk alleles an individual carries. Despite methodological concerns about construct, content, and criterion validity of PRS [1], many studies have shown that PRSs can predict disease status in research-based case-control studies [2–4]. More convincingly, the prediction is also valid in population-based cohort studies and in electronic health record-based studies [5–7].

In this review, we consider how polygenic risk scores may be informative at different points in the disease trajectory, from unaffected individuals being tested for future disease risk to diagnosed patients, assessing how genetic information might inform their treatment or provide prognostic information on disease course. For each stage, we illustrate how PRS might be used and give examples of the current progress in the field.

## Properties of polygenic risk scores

**Fig. 1:**
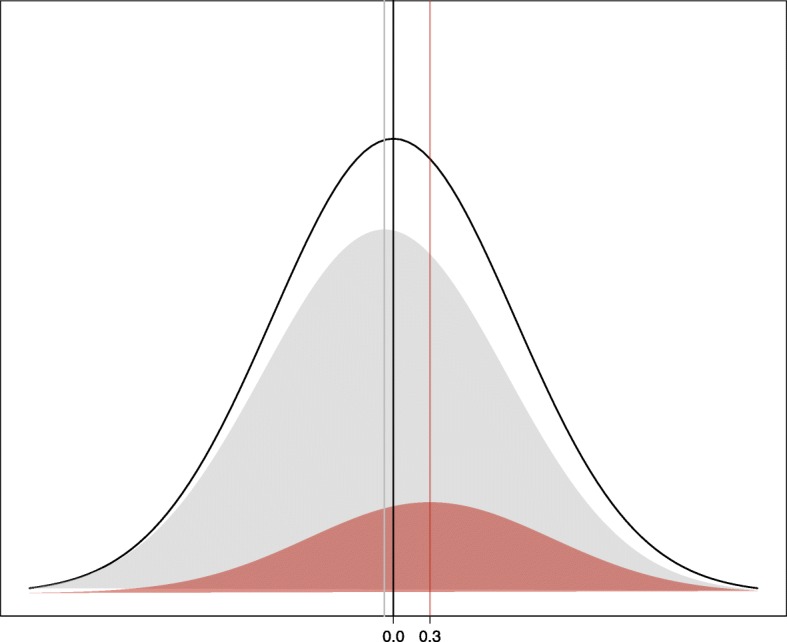
Normal distribution of polygenic risk scores, for a disorder of prevalence 20% (prev), with cases having a mean PRS of *t* = 0.3. Black line: population N(0,1) distribution. Grey shaded area: controls, unaffected with disorder, with mean PRS = − prev × *t*/(1 − prev) = − 0.075. Red shaded area: cases, mean PRS *t* = 0.3. AUC = 0.605, calculated from *Φ* (Cohen’s *d*/√2), where *Φ* is the normal distribution cumulative distribution function, and Cohen’s *d* is the difference between mean PRSs for cases and controls [8]

The PRS is formed from a set of independent risk variants associated with a disorder, based on the current evidence from the largest or most informative genome-wide association studies. For each individual, the number of risk alleles carried at each variant (0, 1, or 2) is summed, weighted by its effect size (i.e. log (OR) for binary traits or beta coefficient for continuous traits). The outcome is a single score of each individual’s genetic loading for a disease or for a continuous trait (Fig. 1).

Summing across variants assumes an additive genetic architecture, with independence of risk variants. Although simplistic, this reflects our best estimate of genetic architecture of common complex disorders, where little evidence of interaction between genetic variants is detected. These additive polygenic risk scores do not model any gene-gene or gene-environment interactions [9]; however, the largest meta-analysis of heritability from twin studies supports a simple additive model in most of the traits examined [10].

Several methods can be used to calculate polygenic risk scores. These include ‘clumping/pruning and thresholding’ methods, where a reduced set of genetic variants is identified through pruning on linkage disequilibrium, and accounting for evidence of association with the trait being studied (clumping). Polygenic risk scores are then calculated summing over all SNPs meeting a *p* value threshold, or set of thresholds, as implemented in PRSice [11] and PLINK [12]. In contrast, other methods assess the best prediction genome-wide by explicitly modelling the correlation structure between variants without attempting to identify a minimal subset of SNPs for prediction; the most widely used implementation is the Bayesian LDpred approach [13]. Many novel risk score methods are under development and may have increased power in comparison with our current methods (for example, SBayesR [14]). We will use the term ‘polygenic risk scores’ to cover all methods that sum genetic data to provide individual risk measures and will assume that these are transformed to have a standard normal distribution. The measures used to assess the predictive ability of a PRS are summarised in Table 1.

**Table 1:** Assessing the clinical utility of polygenic risk scores

A: Population level	
The predictive ability of polygenic risk scores can be measured in research studies, where differences between cases and controls (Fig. 1) or of a continuous trait in a population are assessed. Here, the disease status or trait is pre-established, and the studies measure the extent to which this is determined by the PRS. Outcome measures from such studies include: (1) *R*^2^ from linear regression, which quantifies the proportion of variance in a continuous trait captured by the PRS, or equivalently Nagelkerke’s *R*^2^ for logistic regression for case-control disease status.	
(2) *R*^2^ on a liability scale, which transforms Nagelkerke’s *R*^2^ to reflect disease prevalence, instead of the case-control ratio of the research study [15].	
(3) The area under the receiver operating characteristic curve (AUC) [16], which takes a value from 0.5 to 1. This gives an overall summary of the predictive ability of the model. It is most easily interpreted as the probability that a randomly selected case will have a higher polygenic risk score than a randomly selected control. Such models can also include risk factors such as age and sex, which will increase the AUC values above that based on PRS alone.	
(4) The proportion of the population that has a *k*-fold increased odds (*k* = 2, 3, …), compared to the population disease risk.	
(5) Odds ratio of disease risk conferred by a 1-standard deviation increase in PRS.	
(6) Odds ratio of disease for an individual in the top PRS decile (or other quantiles) compared to individuals in a different part of the PRS distribution. The high-risk group may be compared to the lowest decile, a mid-quintile (e.g. 40–60%), or those outside the high-risk group (0–90%). Comparing the upper and lower tails maximises the odds ratio for impact but raises concerns about the arbitrariness of the quantile used.	
B: Individual level	
In a clinical setting, the focus is on a single person: what information does their PRS give about their risk of disease? Possible outcome measures that are relevant at an individual level include:	
(a) At what percentile in the distribution of PRS does this individual lie? This is between 0 and 100%, with scores having a normal distribution.	
(b) What is this person’s relative risk of disease compared to the average risk in the population?	
(c) What is this person’s absolute risk of disease, and by what age [17]?	

The PRS measure is beguiling in its simplicity, but it is limited in its ability to capture the full genetic loading for a disorder. We currently have an incomplete list of genetic variants associated with a disorder, and the effect sizes used to construct the score are imprecise. The use of tagging SNPs in place of the (unknown) causal variant or variants also limits precision, but a novel methodology has been developed, for example, extensions of the LDpred, in order to address this issue [18, 19]. Further, it is becoming clear that genetic risk scores also capture information from the environment. Evidence for this is seen in family-based studies which show that contributions from the PRS computed from non-transmitted alleles of parents also affect offspring phenotypes. This ‘genetic nurture’ phenomenon indicates, for example, that educational attainment is influenced by both offspring genetics and the non-transmitted parental genetics, which may determine the family environment [20].

There are four important considerations of the information content of a polygenic score, and how it can be interpreted:

1. The known information, which shows where an individual lies compared to others on the risk scale
2. The unknown information from incomplete genetics or unmodelled environment
3. The potential for incorrect information, for example, where the individual differs from characteristics of the research study used to estimate the effect size of each genetic variant by genetic ancestry, age, environmental load, or disease definition, or where there is a technical bias in data collection
4. The intended use of the PRS, for example, more complete information would be required for justifying a pharmacological intervention than for using the PRS to motivate behaviour change

The first two properties of known and unknown information are summarised by the proportion of disease liability that the polygenic risk score captures, whilst our understanding of the incorrect information is still evolving.

## Applicability of PRS across ethnic groups

One of the most challenging aspects of moving PRS to the clinical arena is ensuring that they are equally applicable to all health care users across ethnic groups to limit exacerbating health disparities [21]. This is an important issue both for minority ethnic groups within high-income countries, who may be under-represented in research studies, and for low- and middle-income countries, where genetic studies of the relevant ancestry may not exist because of limited research infrastructure. Current PRS methods rely on an individual’s genetic ancestry being similar to the large GWAS study from which reference effect sizes are taken for PRS calculation and may require access to an ancestry-matched genotype-level reference panel. Such studies are currently only widely available in European ancestries [22, 23], so polygenic risk scores are applicable to only a small proportion of the world’s population; in this paper, unless otherwise stated, study participants are of European ancestries. Transferability of PRS across populations is limited, with PRS generated from GWAS in one population usually providing attenuated predictive accuracy in other populations [21, 24]. Reasons for this include the use of tagging SNPs, differences in the patterns of linkage disequilibrium between populations, and SNP arrays biased to variants of European descent [25]. More importantly, differential genetic drift can cause unpredictable biases when scores inferred from one population are applied in another [26]. At an individual level, it is crucially important that an individual’s PRS is compared to a population-specific distribution so that the interpretation is valid. Progress in performing GWAS on non-European ancestries has been slow, with, for example, < 3% of study participants in the GWAS Catalog were of African ancestry [22]. Large-scale GWAS of diabetes and schizophrenia have been performed in African and East Asian populations [27–29], and novel initiatives of the collection in worldwide populations like the Human Heredity and Health in Africa (H3Africa) Initiative (https://h3africa.org/) and the African Mental Health Research Initiative (https://amari-africa.org) are underway. Key methodological considerations for GWAS in ancestrally diverse populations have been recently discussed, including the choice between performing a meta-analysis stratified by ethnic groups and performing a joint mixed-model across all participants [23]. Novel methods for ‘polyethnic’ scores, like XP-BLUP and Multi-ethnic PRS, which improve predictive accuracy by combining transethnic with ethnic-specific information, are being developed [30–32].

Substantial investment will be needed to achieve the equivalence of genetic information required for equity of access when polygenic risk scores are applied in the clinic [21, 33].

## Clinical utility of PRS

The potential value of polygenic scores is supported by the increasing number of research studies that show a highly significant association between PRS and disease status, but their clinical utility has yet to be established. Can PRS be used by clinicians for disease prediction or stratification, either now or in the future? For this to be achieved, the focus must shift from association with case-control status to the information in the PRS for a single individual. Furthermore, to translate PRS to clinical tools, relative risks that compare individuals across the PRS continuum with a baseline group will eventually need to be transformed to absolute risks for the disease [34, 35]. Unlike monogenic disorders caused by high-penetrance mutations, in complex disorders, the discriminative ability of PRS is compromised by the multifactorial contributors to the disease, the imperfect measurement of the full genetic signal, and the potentially incorrect measurement.

Risk prediction models, including a combination of clinical, biochemistry, lifestyle, and historical risk factors, are currently used to predict 10-year risk of cardiovascular disease and diabetes [36–39]. These models combining risk factors achieve a good prediction (AUCs of 80–85%) and are included in clinical guidelines for prevention and public health [40]. Polygenic risk scores have much lower AUCs, as expected from a single risk factor, and should not be considered as an alternative to these clinical risk models but as a possible addition. With the established polygenic architecture of complex disorders, the improvement of genetic and statistical methodology, and the increase of global genotyped samples, it is reasonable to anticipate that genetic prediction will improve. In the meantime, it may be timely to consider the use of PRS in specific cohorts where there is a higher prior probability of disease.

**Fig. 2:**
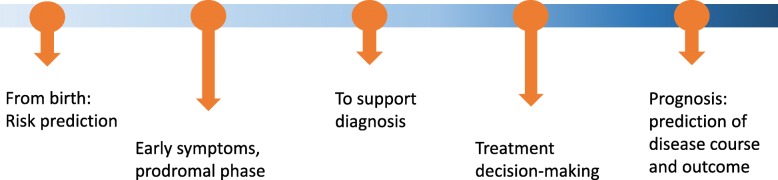
Lifeline of the potential relevance of polygenic risk scores showing points through disease trajectory where polygenic risk scores have the potential to impact clinical care

The current focus is on identifying individuals at high genetic risk of disease for risk stratification. This information could be useful in decisions about participation in screening programmes, lifestyle modifications, or preventive treatment, when available and appropriate. PRS may also be relevant at different points along disease diagnosis and course (Fig. 2). An important consideration is the need to avoid presenting a false impression of genetic determinism (the notion that genes alone define biology). This could otherwise detrimentally impact personal choices, harming physical and mental well-being (e.g. diet, exercise, lifestyle), and possibly even education, employment, or family planning. Research on measuring the beliefs of the public in genetic determinism [41] should expand from single-gene disorders to include polygenic prediction of complex diseases. The widespread interest in PRS is illustrated by their use by direct-to-consumer genetic testing companies; for example, 23andMe now offers polygenic risk scores for T2D. The Polygenic Score Catalog (http://pgscatalog.org/) curates data and tables extracted from polygenic risk scores for common disorders, capturing performance metrics of the PRS developed.

## Disease risk prediction

Although one’s genetic liability is fixed from conception, the risk arising from one’s genes is dynamic, depending on changing factors such as age, environmental exposures, and previous illnesses. For example, if someone is at high genetic risk for alcohol/drug dependence but is never exposed to alcohol or drugs, the genetic risk is irrelevant. Even if sequencing at birth were to become standard clinical practice [42], communicating risk scores at birth is neither appropriate nor useful, and it is likely that genetic data would be stored and interrogated throughout life for both single-gene Mendelian disorders and common polygenic disorders. The decision to assess PRS might be triggered by age, onset of symptoms, family history, or presence of relevant environmental factors. The role that PRS will play in clinical care is currently unclear, and any use of PRS must be predicated on clear clinical utility, with a specific outcome activated by the score. For example, a PRS for coronary artery disease assessed in early adulthood may be useful to encourage healthy behaviour throughout life, although we still lack experience of how to use genetic data to motivate behaviour change [43–45]. Not all preventive strategies are so benign; pharmacological interventions or surgical procedures are more controversial. For example, it would be very difficult to consider prophylactic mastectomy for breast cancer prevention. Even simple decisions like increased screening may result in false positives with significant economic cost to society and unnecessary stress of the individual. At its simplest, PRS may be used to estimate an individual’s lifetime risk of disease. This application follows the design of most genome-wide association studies, differentiating between cases and controls. From the studies performed in different clinical areas assessing the predictive ability of PRS, we discuss below the strongest evidence for potential clinical utility, with special reference to our research area, psychiatric disorders [46].

### Medical conditions

Much of the research to motivate moving polygenic risk scores from research studies to clinical implementation comes from cardiovascular disease, type 2 diabetes, breast and prostate cancers, and Alzheimer’s disease [47]. Khera et al. [2] recently demonstrated in the UK Biobank that PRS can identify which percentage of the sample have at least 3-fold increased risk for coronary artery disease, atrial fibrillation, type 2 diabetes, inflammatory bowel disease, and breast cancer, with the proportion of individuals identified varying between 1.5 and 8% depending on the disorder. Although these effects appear modest, PRS can identify substantial larger fractions of the population at high disease risk than monogenic mutations, making PRS potentially more clinically relevant. Apart from the generic prediction of case-control status, specific applications of PRS have been proposed. For example, the PRSs of left ventricular cardiovascular magnetic resonance phenotypes are predictive of heart failure events independently of clinical risk factors in the UK Biobank [48], whilst elevated genetic score for albuminuria is strongly associated with increased risk of hypertension [49].

In breast cancer, pioneering work in risk prediction, modelling genetic susceptibility based on two genes, *BRCA1* and *BRCA2*, [50] has been expanded to the use of polygenic scores, which have improved predictive ability. In a recent study, a PRS based on 303 genetic variants had an AUC of 0.63, with an odds ratio of 1.61 (95% confidence interval, 1.57–1.65) per unit increase in PRS [3]. Whilst these figures are modest, they translate into a substantial spread of risk in the population: women in the top 1% of PRS have a 4-fold increased risk of developing ER-positive breast cancer and a corresponding 6-fold decreased risk for those in the lowest 1% PRS (both compared to women in the mid-quintile of the PRS distribution). Despite the modest discriminative ability, PRS could be utilised in improving screening programmes, including defining the age at which breast cancer screening should start and the screening interval. In the UK, mammogram screening is offered to women over age 47, when the average 10-year risk of breast cancer is ~ 2.6%. However, Mavaddat et al. [3] show that PRS is informative in stratifying risk, with the 20% of women with the highest polygenic risk scores reaching this level of risk before age 40 and the 20% of women with the lowest scores never reaching this level of risk. This study shows that breast cancer polygenic risk scores already capture sufficient information to identify a high-risk subgroup of women who could be offered mammogram screening at an earlier age. Commercial breast cancer risk tests based on polygenic risk scores are already offered by Myriad Genetics (riskScore™) and Ambry Genetics (AmbryScore).

These PRS studies were all performed in European-ancestry populations, and expansion to worldwide populations is essential. For example, a study of lung cancer-associated variants in Chinese populations [51] demonstrated that PRS was an independent risk stratification indicator for lung cancer beyond age and smoking pack-years. Polygenic risk scores have been less widely applied to auto-immune disorders. In type 1 diabetes, strong genetic risk scores have been created, with the large effect of the HLA haplotypes increasing AUC values to > 0.8 in European ancestry and Hispanic populations [52]. An ancestry-specific genetic score using 7 SNPs outperformed a European-based genetic score in African ancestry participants (AUC 0.87 and 0.80, respectively) [53].

PRS also can be used to predict continuous traits in the population like BMI, which is important as a risk factor for cardiometabolic traits. A recent large study showed that participants in the highest BMI PRS decile have a BMI that is 2.6 kg/m^2^ higher than those in the mid-quantile PRS—to put in context, this is half the width of the ‘overweight category’ of BMI (from 25 to 29.9 mg/m^2^)—and corresponds to 31% of individuals with a BMI > 40 (obesity class 3) [54]. Genetically dissected trajectories of BMI across the lifespan help us identify where genetic prediction starts to be relevant. BMI PRS is only minimally associated with birthweight; it has increasing prediction through childhood, and by age 18, the differences in BMI by PRS quartile are similar to that seen in adulthood [54]. Despite the significant polygenic prediction of BMI in the general population, it remains to be determined whether BMI PRS has clinical utility in high-risk populations. For example, a common adverse effect of antipsychotics or antidepressants is weight gain; however, we do not know whether this is determined or modified by the BMI PRS.

### Psychiatric disorders

Polygenic risk scores for major depression can be calculated using results from recent genome-wide association studies [4, 55]. Wray et al. identified 44 SNPs at genome-wide significance and showed that a PRS built from SNPs with a *p* value < 0.05 had the highest predictive ability. Individuals in the upper PRS decile had approximately 2.5-fold increased risk of disease compared to those in the lowest decile, which translates into a substantial change in absolute risk, given an approximate 15% lifetime risk of major depression. However, this score has an AUC of 0.57 and captures only 2% of variance in disease risk (*R*^2^ on the liability scale), whilst the remaining 98% is uncaptured by the PRS. An individual’s risk of depression therefore comprises 2% of measurable genetic risk score and 98% unaccounted variation from unmodelled genetic and environmental factors. Even for an individual at very high genetic risk, the PRS signal would be overpowered by the unmodelled component. Depression PRS is therefore not yet useful, and any future utility would be based on substantially increasing the variance explained by the PRS or by joint modelling of genetic and environmental risk factors. Increasing the sample size with the inclusion of broader self-reported definitions of depression [55] resulted in a modest increase of the variance explained by PRS, albeit at the cost of specificity for major depression. Phenotypic refinement has been proposed as an alternative to produce more clinically relevant findings [56].

For schizophrenia, the predictive ability is higher, with the current score accounting for 7% of trait variance and an AUC of 0.61, but these values are still far below that needed for an individual’s score to have sufficient signal for interpretation or for clinical utility [57]. There is greater potential for using risk prediction from genetics in schizophrenia, since the heritability of 65–80% [58, 59] is much higher than 37% for major depression [60], but the substantially different disease lifetime risks (< 1% for schizophrenia vs. 15% for major depressive disorder) is also relevant. Even though polygenic scores are not meaningful for general prediction [57], there are points in the clinical care pathway where PRS could be useful in achieving an earlier or a more precise diagnosis. For example, in first-episode psychosis, we have shown that schizophrenia PRS can differentiate schizophrenia from other psychosis diagnoses (Nagelkerke’s *R*^2^ of 9% and those in the top quintile of PRS having an approximately 2-fold increased risk of being subsequently diagnosed with schizophrenia) [24]. This is a low predictive ability, but the setting within first-episode psychosis cases makes it more appealing because (1) it does not require genotyping of the general population, only people with psychosis, and (2) it is not relevant to major decisions (like treat/not treat), but could provide additional information potentially useful for the care plan. In addition to assisting diagnosis, genotype data could be used to calculate other PRS in secondary screening, for example, cardiovascular disease, since psychosis cases are already at high cardiometabolic risk.

In Alzheimer’s disease (AD), the three *APOE* variants (ε2, ε3, and ε4) have been consistently associated with disease risk, making it the strongest single-gene predictor at a population level in neuropsychiatry. However, additional risk variants summarised in a PRS improve the prediction model further. For example, Desikan et al. [61] showed that amongst *APOE* ε3/3 carriers, PRS modified the expected age of AD onset by more than 10 years between the lowest and highest deciles (hazard ratio 3.34, *p* = 10^−22^).

## PRS by environment interaction

All the common disorders considered here have both genetic and environmental risk factors. Another area of exploration in PRS is possible gene-environment interaction, which implies that the effect of the disease PRS would depend on the level of the environmental risk factor. This interaction model contrasts with an additive model, where PRS and environment contribute independently to disease risk. With a positive interaction, the effect of a high PRS would be amplified in the presence of an environmental risk factor (E), putting this subgroup of the population (G+, E+) at particularly high risk of disease. These individuals could form a specific target group for interventions, identified by either PRS or environment, or both.

Despite the attraction of identifying PRS-E interactions, currently, there is no strong evidence supporting these interaction models.

In depression, childhood maltreatment is an important risk factor for later diagnosis with depression. A meta-analysis of 6000 individuals confirmed strong effects for both PRS and childhood maltreatment contributing to the risk of depression but showed no evidence of an interaction [62]. In cardiovascular disease, an extensive study of lifestyle effects (diet, exercise, BMI, smoking) with polygenic risk scores showed strong effects from both sources, but no evidence of an interaction [63]. Similarly, a recent cohort study of UK Biobank participants aged > 60 without cognitive impairment, followed up for 8 years, both genetic risk and lifestyle factors predicted incidence of dementia, but no interaction was found [64]. Even though this paucity of confirmed interactions in large samples is not helpful in identifying individuals at very high risk, testing and disproving interactions are essential for correct joint modelling of genes and environment for risk prediction [65].

## PRS in treatment choice

Pharmacogenetic studies test how genetic variants affect response to treatment, with the aim of assisting treatment choices to maximise efficacy and minimise side effects. Most progress has been made in identifying rare high-risk variants that increase risk of adverse drug events (for example, abacavir and HLA-B*57:01, carbamazepine and HLA-B*15:02), whilst prediction of treatment efficacy has largely evaded genetic dissection.

The potential impact of PRS in treatment response is unknown, but an easy first target is to test whether genetic disease susceptibility also plays a role in treatment outcome. Currently, the strongest evidence for a role of PRS in treatment response is in statin use to reduce the risk of first coronary event, where studies have shown that the relative risk reduction is higher in those at high genetic risk for cardiovascular disease [66, 67]. These results are in line with the previous reporting of better efficacy of statins in high-risk samples, for example, due to diabetes, hypertension, or high CRP concentrations [68]. A recent study demonstrated a potential role of PRS for electrocardiogram parameters in predicting the cardiac electrical response to sodium channel blockade [69].

In psychiatric disorders, only weak evidence exists to suggest that the PRS for disorder susceptibility might be predictive of treatment response in depression [70, 71] or psychosis [72]. Further studies to identify specific treatment response polygenic risk scores are in progress in these disorders, but it is challenging to achieve sufficiently large sample sizes, with accurately captured response measures. Meta-analysis studies are underway, pooling clinical trials and observational studies of response to anti-depressants and to anti-psychotics. These would identify polygenic predictors for treatment response that might be useful in, for example, deciding between pharmacological and psychological treatment for depression [73]. Only one third of patients respond to the first anti-depressant prescribed [74], so a polygenic predictor might be useful to guide treatment; even a modest increase in the proportion of patients responding could have a substantial impact on the effectiveness and time to recovery. An important perspective in genetic testing for treatment response is in identifying patients who are unlikely to respond to a specific drug, as Gibson highlights [75, 76]. This could reduce the time taken by clinicians to find efficacious treatment, improve treatment response, and prevent treatment-related adverse effects, which is cost-effective for both the patient and the healthcare system. When the choice of treatment is not dictated by different effectiveness, but from personal experience, preference, or intuition on a trial-and-error basis, PRS can potentially give some quantifiable information to be considered along other lines of evidence.

## PRS to refine penetrance of high-risk variants

Evidence is accruing that polygenic risk scores have a role in both the general population and carriers of rare, high-risk genetic variants. In disorders as diverse as breast cancer, developmental disorders, and schizophrenia, polygenic risk scores affect penetrance, acting as moderators for high-risk variants or structural variation. This highlights a possible role for PRS within the well-established framework of high-risk genetic testing. For example, the Deciphering Developmental Disorders study showed that in 7000 children with severe neurodevelopmental disorders expected to be monogenic in aetiology, common variation affects the overall risk of severe neurodevelopmental disorders. It explains over 7% of the variance and affects the individual presentation of symptoms [77]. The role of common variation in moderating expressivity was confirmed in a large electronic health record study, where, in addition to the large effect from the rare pathogenic variants, PRSs for height and BMI were associated with clinical outcome [78]. In breast cancer, the absolute risk increase in carriers of *BRCA1* and *BRCA2* pathogenic variants depends on breast cancer polygenic risk scores, which might influence clinical decision-making [79]. The joint modelling of common and rare variants for breast cancer risk prediction can now be performed in the risk calculation tool, BOADICEA [80]. Similarly, in schizophrenia, both structural variation and PRS contribute to risk: schizophrenia cases that carry confirmed copy number variants (CNVs) have higher PRS than cases which do not; within carriers of CNVs, schizophrenia cases have higher PRS than controls [81, 82]. Hence, even in the presence of CNVs with high penetrance [83], polygenic scores affect the overall risk of disease and may be relevant to the clinical expression in CNVs associated with multiple phenotypes like the 22q11.2 deletion [84].

## Role of direct-to-consumer testing

Direct-to-consumer (DTC) genetic testing companies give consumers easy access to their genetic data, specifically genotyping on genome-wide chips of up to 1 million variants. Estimates suggest that 26 million people had used online DTC companies such as Ancestry.com and 23andMe up to the end of 2018 (https://www.technologyreview.com/s/612880/more-than-26-million-people-have-taken-an-at-home-ancestry-test/). Whilst many purchasers are initially interested in ancestry testing, customers may then move on to analyse their genetic data for health [85], downloading their raw genotype data to explore in third-party interpretation programmes. These programmes are unregulated and differ in the genetic risks provided, the explanatory information provided, and the cautions given over interpretation. Some sites allow users to calculate polygenic risk scores; for example, Impute.me (https://www.impute.me/) shows users where their polygenic risk score lies against a population-specific distribution of scores. Allelica provides an online service calculating polygenic risk scores [86]. In direct-to-consumer genetic testing, MyHeritage (https://www.myheritage.com/health/genetic-risk-reports) provides polygenic risk scores on four traits, ‘for people who are of mainly European ancestry’. The most detailed assessment of PRS in a DTC setting is from 23andMe, whose white paper presents their epidemiological modelling and the challenges of deriving individual-level absolute disease risks from PRS [67]. 23andMe provides polygenic risk scores for type 2 diabetes; based on external validation, their models have AUC values of between 59 and 65%, similar to those obtained from research studies [87]. Their customer reports give an estimate of the remaining lifetime risk of T2D based on genetics, age, and ancestry, with additional information on how BMI, diet, and exercise habits affect T2D prevalence.

The accuracy and generalisability of any PRS model need to be validated with external data, but even when the scientific basis is robust, the correct, unbiased interpretation of risk profiles by the consumers will need to be evaluated. The extent to which DTC genetic testing will move polygenic risk scores into the clinical arena is unknown. For example, patients bringing their DTC results may motivate conversations between primary care physicians and patients on health education.

## Conclusions and future directions

Polygenic risk scores have moved from research discovery studies to clinical research studies (for example, a trial aiming to assess the impact of PRS reporting on breast cancer risk management recommendations NCT03688204) (https://clinicaltrials.gov/ct2/show/NCT03688204) and have started on the slow path to clinical implementation. This review discusses some of the disorders where this is likely to occur and highlights the obstacles that remain in harnessing the information contained in PRS. The strongest evidence for PRS currently comes from cardiovascular diseases and breast cancer, where risk stratification of those at high polygenic risk has clinical utility [2, 3, 47]. Other disorders are likely to follow; however, there is still a long route to be covered before PRSs become useful tools for clinicians (Table 2).

**Table 2:** A brief overview of the steps required to make PRS relevant in a clinical setting

1. Realistic estimation of predictive ability in clinical populations, which may differ from research samples in disease severity, ancestral diversity, and exposure to environmental risk	
2. Identification of the intended purpose of the PRS, which may affect its design and validation, and relevant clinical questions that can be answered, for example, prediction of severity, course of illness, or response to treatment	
3. Recognition that even though not useful for the majority of the population with PRS in the middle of the distribution, the outcome may be relevant for those with high or low PRS, in the tails of the distribution	
4. Clarification if PRS has an additive or interaction effect with established epidemiological or biological risk factors before combining in joint prediction models [88]	
5. Engagement of clinicians and service users, to ensure that any application of polygenic risk scores avoids deterministic interpretations and is based on the understanding that PRS is an indicator, not a precise measure	

One challenge of exploring the value of PRS within the clinical setting to predict the outcome, or determine the treatment, is that the sample sizes from case-only clinical studies with relevant phenotypic data related to the course of illness, treatment response, or adverse effects are substantially lower than those from case-control disease susceptibility studies. The latter requires minimal phenotypic information—a clinical diagnosis, or self-report—whilst determining prognosis or treatment outcome requires longitudinal follow-up across sustained periods of time. This is expensive and challenging to collect, and such studies often have much smaller sample sizes. Electronic health records (EHR) may provide longitudinal data, but ‘treatment response’ is often poorly recorded and needs to be captured laterally through prescription records. We highlight how applying PRS in treatment response may better facilitate clinical utility, as the genetic data will complement the clinician’s choice of treatment. We envisage that the role of PRS in informing treatment choices, for example, prioritising pharmaceutical or psychosocial interventions or providing quantitative information on the benefit to harm ratio for each treatment, rather than treat/not treat decisions, may be the low-hanging fruit where the clinical utility of PRS will become apparent.

An ultimate goal might be to have genotype data—and later whole-genome sequence data—integrated into our clinical record; this could then be interrogated at each clinical encounter for relevant information on risk prediction, treatment response, and disorder prognosis. For polygenic risk scores, this is not yet scientifically justified and is technically challenging, particularly since an individual score must be built and interpreted against the appropriate genomic reference population, which may not be available. Both these restrictions are likely to change with continuing scientific progress in uncovering the genetic contribution to common diseases and with expanding capabilities of electronic health records [7]. Projects such as the eMERGE network (https://emerge-network.org/) are leading the way in these initiatives, although also highlighting clinicians’ concerns about the role of unsolicited genetic results in their practice [89].

We focus on two limitations to the implementation of polygenic risk scores in clinical practice: firstly, the weaker evidence base in application to non-European ancestry; this needs substantial research investment in study collection worldwide and in methodological research to improve genetic prediction in admixed individuals. Secondly, major challenges exist with the interpretation of polygenic risk scores. At its simplest, an individual can be placed on the distribution, ‘your PRS lies at the 22.8th percentile’, which gives limited information on their lifetime risk. But a more nuanced interpretation is needed, for example, a lifetime risk of disease that combines genetic information with their current age, sex, and environmental and clinical risk factors.

In summary, we have made astounding biological advances in uncovering the genetic component to common complex disorders since the advent of genome-wide association studies in 2007. This is slowly moving from research discovery to clinical implementation, but much work remains in acquiring the necessary research base for polygenic risk scores and in establishing how the information can be best be used and communicated.

## Data Availability

Not applicable

## Abbreviations

AUC: Area under the receiver operating characteristic curve
BMI: Body mass index
CNV: Copy number variant
DTC: Direct-to-consumer
EHR: Electronic health records
OR: Odds ratio
PRS: Polygenic risk score
